# Intravitreal Injections for Macular Edema Secondary to Retinal Vein Occlusion: Long-Term Functional and Anatomic Outcomes

**DOI:** 10.1155/2020/7817542

**Published:** 2020-02-13

**Authors:** Emilia Maggio, Maurizio Mete, Giorgia Maraone, Marcella Attanasio, Massimo Guerriero, Grazia Pertile

**Affiliations:** ^1^IRCCS Sacro Cuore Don Calabria Hospital, Via Don Sempreboni 5-Negrar 37024, Verona, Italy; ^2^Department Computer Science, University of Verona, Strada le Grazie 15-37134, Verona, Italy

## Abstract

**Purpose:**

To report the long-term visual and anatomic outcomes of intravitreal injections for macular edema (ME) secondary to retinal vein occlusion (RVO) in a real-life clinical setting.

**Design:**

Retrospective interventional case series.

**Methods:**

A total of 223 consecutive eyes with ME secondary to RVO, treated with the first three intravitreal Ranibizumab or dexamethasone injections between August 2008 and September 2018, were enrolled in the study. Subsequent retreatment was guided by best-corrected visual acuity (BCVA) and central macular thickness (CMT) measurements, aimed at achieving macular fluid regression and BCVA stability. BCVA and CMT were recorded at baseline and at subsequent annual time points. The mean number of injections administered each year and the incidence of adverse events were recorded.

**Results:**

The mean BCVA and CMT at baseline were 0.79 logMar (SD 0.71) and 615.7 *μ*m (SD 257.5), respectively. The mean follow-up (FU) period was 47.8 months (min 12–max 120). At 12 months, the mean BCVA and CMT had significantly improved to 0.62 logMar (SD 0.68; *p* < 0.0001) and 401.04 *μ*m (SD 257.5), respectively. The mean follow-up (FU) period was 47.8 months (min 12–max 120). At 12 months, the mean BCVA and CMT had significantly improved to 0.62 logMar (SD 0.68; *p* < 0.0001) and 401.04

**Conclusion:**

Intravitreal Ranibizumab and/or dexamethasone injections were found to be effective at inducing a long-lasting improvement of BCVA and CMT in a real-life clinical setting. A safety profile similar to that already well-established in Ranibizumab and dexamethasone treatment was observed, as well as a steady decrease in the number of intraocular injections required. The results support intravitreal treatments for BRVO and CRVO in patient populations with similar characteristics in similar settings.

## 1. Introduction

Retinal vein occlusion (RVO) is the second most common cause of vision loss due to retinal vascular disease, after diabetic retinopathy [1]. Macular edema (ME) is a frequent and sight-threatening complication of both central (CRVO) and branch (BRVO) retinal vein occlusion [2, 3].

In the past, treatment options for ME secondary to RVO were limited. The CRVO study group [4] demonstrated that grid laser photocoagulation is not effective in cases of visual impairment due to CRVO-related ME, while the BRVO study group [5] reported its efficacy in treating ME secondary to BRVO. Since the publication of those two reports, the standard of care for BRVO-related ME became grid laser photocoagulation and for CRVO-related ME was observation. However, evidence from subsequent randomized controlled trials has demonstrated significant visual and anatomic improvements among patients with either CRVO- or BRVO-related ME who were treated with intravitreal injections of vascular endothelial growth factor (VEGF) inhibitors or with corticosteroids.

In particular, the Geneva study group [6], in a randomized, sham-controlled, clinical trial conducted on 1267 patients, found significantly greater improvement in the mean best-corrected visual acuity (BCVA) of eyes treated with dexamethasone intravitreal implant (DEX implant; Ozurdex^®^, Allergan, Inc., Irvine, CA, USA) compared to controls, with a good safety profile. Significant visual and anatomic improvements among patients receiving VEGF inhibitors have also been demonstrated in randomized clinical studies including COPERNICUS, GALILEO, BRAVO, CRUISE, and VIBRANT [7–11]. The CRUISE study reported a mean gain in BCVA of 13.9 letters in CRVO eyes at 12 months. The BRAVO study, with a similar design to CRUISE, demonstrated mean BCVA improvements by 16.4 and 18.3 letters in the 0.3 and 0.5 mg groups, respectively, among BRVO eyes, over the 12-month study period. Extension studies following BRAVO and CRUISE [12] have given some insight into the outcomes of anti-VEGF therapy for RVO up to 4 years after initiating treatment.

In light of these favourable results, patient and physician expectations in the visual outcomes with intravitreal injections for RVO-related ME have increased greatly. However, results from clinical trials might differ considerably from those found in real-world settings, given that the intensive treatment schedules and close monitoring, typically employed in clinical trials, are very difficult to replicate in real-life. Moreover, the strict eligibility criteria of trials may result in selected populations that do not represent those routinely found in clinical practice.

Although intravitreal injection therapy has now become the treatment of choice for RVO-related ME in many countries, there is very limited data available on the long-term outcomes in real-world settings.

The aim of this study was to investigate the long-term visual and anatomic outcomes in patients with ME secondary to RVO treated with intravitreal injections of Ranibizumab and/or dexamethasone in a real-world setting.

## 2. Methods

### 2.1. Study Design

This research is a retrospective interventional case series undertaken at a single tertiary referral center. It evaluated the long-term anatomic and functional outcomes of all consecutive eyes that were (a) diagnosed with recent onset, previously untreated ME secondary to RVO at the IRCCS Sacro Cuore Hospital, Negrar, Verona, Italy, and (b) treated with their first injection between August 2008 and September 2018.

The primary end point was the evaluation of any change in mean BCVA and central macular thickness (CMT) from baseline to the 12-month follow-up (FU) visit and at each subsequent annual FU visit thereafter.

Secondary endpoints were as follows:

- The number of injections received at the end of the first 12-month period and thereafter in the subsequent years of treatment
- The relationship between BCVA and CMT throughout the study period
- The incidence of adverse events
- The influence of the following factors: age, sex, presence of ischemia, type of RVO (BRVO/CRVO), FU duration, and baseline BCVA on visual outcomes

This study complied with Declaration of Helsinki regulations. The IRCCS Sacro Cuore Hospital Institutional Review Board provided approval for the review of patient data.

### 2.2. Study Population

Inclusion criteria were

- age ≥18 years
- ME involving the foveal center secondary to BRVO or CRVO
- CMT ≥ 350 *μ*m
- ME treatment naïve
- recent onset of RVO (less than 6 months since diagnosis)
- a minimum FU of 12 months

When both eyes of a patient met the eligibility criteria, they were both included in the study.

Exclusion criteria were

- any previous treatment with focal/grid laser macular treatment, anti-VEGF, or corticosteroids injection
- the presence of concomitant diseases that could influence outcomes, such as high myopia (>6 D), uveitis history, diabetic retinopathy, and macular holes
- a history of vitreoretinal surgery
- inadequate imaging (i.e., severe media opacities, asteroid hyalosis, and synchysis scintillans)
- ME secondary to diseases other than RVO

### 2.3. Treatment Protocol and Evaluation Procedures

In accordance with the routine practice for patients treated with intravitreal injections at Sacro Cuore Hospital [13], at baseline all patients underwent a complete ophthalmologic examination, including medical history, BCVA evaluation, slit-lamp biomicroscopy, intraocular pressure (IOP) measurement, dilated fundus examination with a 90 diopters indirect lens, optical coherence tomography (spectral domain OCT-SLO Heidelberg Engineering, Heidelberg, Germany) and fluorescein angiography (FA) with the Heidelberg Retina Angiograph (HRA). BCVA was measured by Snellen visual charts and converted to logarithm of the minimum angle of resolution (logMAR) units for statistical analysis.

RVO was classified as nonischemic or ischemic at the initial visit; any conversion of status from nonischemic to ischemic was carefully monitored and evaluated during the FU. Eyes affected by ischemic RVO with evidence of neovascularization, or those at high risk of its development, underwent laser photocoagulation of the areas of peripheral retinal nonperfusion.

Eyes underwent a loading phase with three consecutive monthly intravitreal injections of Ranibizumab. The eyes were then periodically inspected through complete ophthalmological examinations to determine the need for pro re nata (PRN) injections. In particular, the FU examinations included BCVA evaluation, slit-lamp biomicroscopy, IOP evaluation, fundus examination, OCT, and, at the physician's discretion, FA. Retreatment criteria for PRN injections followed a BCVA- and OCT-driven regimen aimed at achieving complete macular fluid regression and BCVA stability. Upon reaching stability, patients were checked bimonthly or quarterly as per physician discretion. Treatment was then continued in the instance of a CMT increase or a BCVA decline due to recurring ME.

Beginning in January 2015, intravitreal dexamethasone implant (Ozurdex) has been at our disposal to treat ME secondary to RVO. Therefore, it was associated with intravitreal Ranibizumab injections in cases of incomplete response to anti-VEGF, as determined by the treating clinician. In general, eyes were considered incomplete responders in case they did not manifest improvement in CMT of at least 20% after a minimum number of six intravitreal anti-VEGF injections. Moreover, Ozurdex was used as a first therapeutic option in pseudophakic eyes with no glaucoma and with no history of IOP increase after topical therapy with corticosteroids. Focal/grid laser macular treatment was also associated with intravitreal injections in cases of incomplete response to anti-VEGF and/or to Ozurdex, as determined by the treating physician. Laser photocoagulation of areas of peripheral retinal nonperfusion and cataract surgery were allowed throughout the study period.

## 3. Statistics

Continuous data were expressed by mean, standard deviation, and min and max value. The two-sample *t*-test for unpaired data was used to compare the means for normal distributed data, while the correspondent nonparametric Wilcoxon rank-sum test was used for nonnormal data. The chi-square test was used to test the statistical association between two categorical variables, while the Pearson linear correlation index was used to evaluate the linear correlation between two continuous variables. A *p* value less than 0.05 was considered for the statistical significance.

Data were analyzed by STATA vers. 15 (StataCorp, 2017, Stata Statistical Software: Release 15, College Station, TX: StataCorp LLC).

## 4. Results

### 4.1. Study Population

A total of 223 eyes from 207 consecutive patients met the inclusion criteria and were enrolled in this study, 99 eyes with CRVO and 124 with BRVO. The mean time period since RVO diagnosis was 1.2 months (SD 2.01; min 0.5–max 6). The mean follow-up was 47.8 months (SD 27.3; min 12–max 120). The baseline characteristics of the study population are summarized in Table 1.

When comparing BRVO and CRVO subgroups at baseline, no significant differences were found in age, sex, or number of phakic/pseudophakic or ischemic/nonischemic eyes. On the contrary, mean BCVA and CMT differed significantly in the subgroups, being significantly worse in CRVO eyes (Table 1; *p* value <0.0001).

Furthermore, mean baseline BCVA and CMT also differed significantly in ischemic and nonischemic RVO eyes (mean baseline BCVA: 0.67 (SD 0.55) logMar for nonischemic versus 0.9 (SD 0.81) for ischemic RVO (*p* value: 0.0065); mean baseline CMT: 561.9 (SD 216.1) *μ*m for nonischemic versus 659.4 (SD 280.2) for ischemic RVO (*p* value: 0.0018)).

### 4.2. Visual and Anatomic Outcomes after Treatment

The mean baseline BCVA in the whole population was 0.79 (SD 0.71). At the 12-month and 2-year FU visits, it significantly improved to 0.62 (SD 0.68; *p* value <0.0001) and 0.63 (SD 0.71; *p* value: 0.009), respectively. Mean BCVA and visual improvements at the subsequent annual time points are reported in Table 2. At each annual time point, the mean BCVA was found to be improved compared to that of baseline, although not statistically significantly so at the 3-year and 4-year follow-up visits. Improvement was significant, however, at subsequent follow-up visits and at the final visit.

Sex, FU duration, and phakic/pseudophakic status did not show any significant influence on visual outcome. On the contrary, greater visual improvement significantly correlated with better baseline BCVA (linear correlation coefficient 0.6226; *p* value <0.0001). Moreover, the presence of ischemia (*p* value = 0.004), older age (*p* value: 0.006), and the type of RVO (CRVO/BRVO) (*p* value: 0.003) was negatively associated with visual outcomes.

At the 12-month FU visit, 35 (15.7%) eyes gained ≥1 line, 170 (76.2%) were stable (less than 1-line gain or loss), 18 (8.1%) lost ≥1 line. The number of eyes gaining/losing ≥1 line at each subsequent annual FU visit is reported in Supplementary Material [Supplementary-material supplementary-material-1].

The mean baseline CMT was 615.7 *μ*m (SD 257.5). It significantly improved to 401.04 *μ*m (SD 183.8) and 376.4 *μ*m (171.2) at the 12-month and 2-year FU visits, respectively. Improvements in CMT were significant over the entire study period up until the final FU visit (Table 2).

### 4.3. BCVA-CMT Correlation

The analysis of correlation between BCVA and CMT showed that a weak correlation was only detectable at baseline and at the first annual time point (at baseline: linear correlation coefficient 0.38; *p* value <0.0001), while no correlation was apparent over the subsequent FU period (at the 5-year FU visit: linear correlation coefficient 0.22; *p* value <0.06).

### 4.4. Comparison between BRVO versus CRVO Outcomes

**Figure 1:**
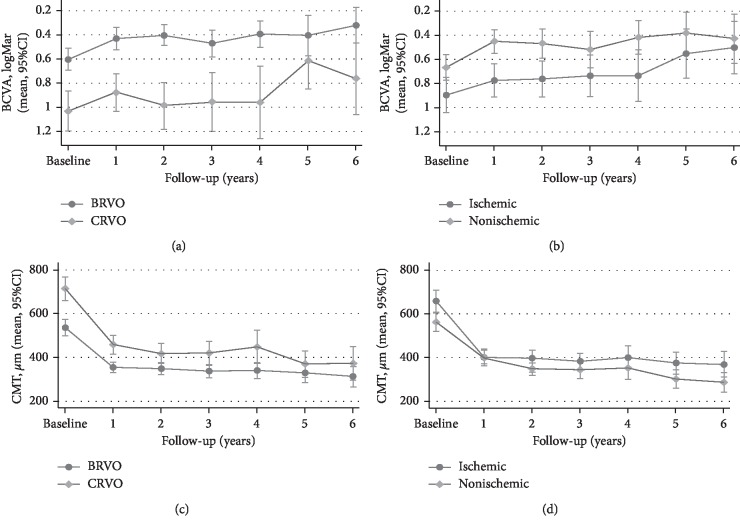
Changes in BCVA and CMT throughout the study period for each subgroup. (a) Changes in BCVA for BRVO/CRVO subgroups. (b) Changes in BCVA for ischemic/nonischemic subgroups. (c) Changes in CMT for BRVO/CRVO subgroups. (d) Changes in CMT for ischemic/nonischemic subgroups.

At the 12-month FU visit, both BRVO and CRVO eyes improved significantly in BCVA and CMT (Figures 1(a) and 1(c)); however, a statistically significant difference in mean BCVA and CMT between the two groups was still detectable.  Table 3 shows mean visual and anatomic outcomes at each time point in BRVO versus CRVO eyes. The mean BCVA was found to be significantly better in BRVO eyes compared to CRVO over the entire FU period; mean CMT was significantly lower in BRVO eyes at the first annual time points, while up until the 5-year follow-up visit, no differences could be found in CMT between BRVO and CRVO eyes.

### 4.5. Ischemic versus Nonischemic RVO Outcomes

When comparing ischemic and nonischemic subgroups at baseline, no significant differences were found in age, sex, number of phakic/pseudophakic eyes, or FU duration. On the contrary, mean BCVA and CMT differed significantly, being worse in ischemic eyes (Figure 1(b); *p* value: 0.0065).

At the 12-month FU visit, mean BCVA significantly improved in both subgroups (Figure 1(b)). However, visual improvements in nonischemic BRVO eyes were significantly higher than those of ischemic eyes, throughout the entire FU period. On the contrary, no significant differences were found in visual outcome when comparing ischemic and nonischemic CRVO eyes (Supplementary Material [Supplementary-material supplementary-material-1]). No significant difference in CMT improvement was found when comparing ischemic and nonischemic subgroups, both for BRVO and CRVO eyes, throughout the entire FU period (Supplementary Material [Supplementary-material supplementary-material-1]).

### 4.6. Number of Injections

The mean number of injections administered in the first year was 4.08 (SD 2.1) for Ranibizumab and 1.5 (SD 0.6) for Ozurdex. The number of injections decreased to a mean number of 3.02 (SD 1.05) for Ranibizumab and 0.62 (SD 0.9) for Ozurdex in the second year. The number of injections administered up until the 6^th^ year is reported in Supplementary Material [Supplementary-material supplementary-material-1]. At the 7-year FU visit, only 11 eyes received additional Ranibizumab injections and 7 eyes received Ozurdex injections. At the 8-year FU visit, 1 eye was treated with Ranibizumab and 3 eyes with Ozurdex.

In the study population 184 eyes were phakic, while the remaining eyes were pseudophakic. Cataract surgery was performed on 81 eyes during the period of investigation. In addition, 82 eyes underwent laser photocoagulation of areas of peripheral retinal nonperfusion and 77 eyes underwent focal/grid macular laser treatment.

### 4.7. Adverse Events

Table 4 summarizes the adverse events that occurred in the study population through the treatment with intravitreal injections.

A transient increase in IOP was found in 65 (29.1%) eyes (min 20, max 38 mmHg); this was treated with topical IOP-lowering medication or kept under observation, with no need for additional procedures. One eye with BRVO developed an increase in IOP that was refractory to topical medications after two Ozurdex injections. It was therefore treated with trabeculectomy. IOP was 38 mmHg and reduced to 10 mmHg after surgery, remaining stable over the entire subsequent FU period. The baseline visual acuity in this eye was 1.01 logMar (20/250); at the end of the study period (43-month FU), BCVA improved to 0.6 logMar (20/80).

One patient with CRVO, a female aged 66 years, developed endophthalmitis two days after the second Ozurdex injection. She was treated with pars plana vitrectomy, phacoemulsification, and intravitreal injection of vancomycin and ceftazidime. The Ozurdex implant was not removed. The treatment resulted in the infection's regression, with no residual vitreous debris detectable upon subsequent examinations. The baseline visual acuity was 0.7 logMar (20/100). At the end of the study period, BCVA had decreased to 1.3 logMar (20/400) as a consequence of the endophthalmitis.

No additional serious adverse events were observed during the FU period, as is reported in Table 4.

## 5. Discussion

In the present study, intravitreal treatment with Ranibizumab and/or dexamethasone was found to effectively provide long-lasting visual and anatomic improvement to eyes affected by RVO that were treated in a real-life clinical setting. In particular, this analysis presents the clinical outcome over a mean FU period of almost 4 years, showing significant visual gains with a flexible dosing regimen and decreasing number of intraocular injections over the period of investigation.

The visual and anatomic outcomes that are achievable with intravitreal treatment for RVO have been described in several randomized clinical trials. However, limited data are available for real-world clinical experiences in large patient populations treated over long FU periods.

Spooner et al. [14] have recently reported their real-life experience with anti-VEGF for RVO-related ME, describing good long-term outcomes in 68 eyes over a 5-year FU period. In our population, the improvement in visual acuity was lower than that reported by Spooner et al. This may be related to the worse baseline visual acuity in our population (conversion to approximate ETDRS letter score [15]: 54.9 versus 61.4 for BRVO; 33.5 versus 54.1 for CRVO) and the greater number of ischemic RVO (55.2% versus 27.9%). This reflects a more difficult-to-treat population with a lower potential for visual recovery. Another real-life analysis over a long FU period (4 years) included only 28 eyes affected by BRVO. In that study, Rezar et al. [16] reported a slightly better outcome compared to that of our population, but, once again, there was a lower number of ischemic BRVO eyes as compared to our study (33% versus 50.8%). It is well known that visual gains after treatment may be strongly influenced by factors such as extent of ischemic macular damage, retinal pigment epithelium atrophic changes, and progressive apoptotic cell death. Accordingly, eyes with ischemic RVO and low baseline visual acuity might experience low or no visual improvement after treatment. However, these conditions are not uncommon in eyes affected by RVO; therefore, it is worthwhile investigating their response to intravitreal treatment. Previous studies [17, 18] found that while a poor final visual acuity is recorded in these eyes if left untreated, significant improvement may be seen with intravitreal treatments over short FU periods. Our results show that significant visual gains are achievable also over longer periods of treatment, despite the more severe baseline condition. This represents an important finding as long-term efficacy is crucial in the treatment of RVO given that the mean age of onset is estimated to be <60 years in about 42% of patients and <70 years in about 72% [1].

The evaluation of possible predictive factors for better visual outcomes revealed only a weak correlation between visual improvement and CMT, which disappeared over the long-term FU period. This highlights that additional factors, other than macular thickness, such as photoreceptor damage and progressive retinal atrophy, may contribute to the impairment in visual acuity. In our population, ischemia and older age were found to be detrimental to visual acuity, while better visual outcomes were detected in eyes with a better baseline BCVA. Furthermore, better visual acuity was recorded in eyes with BRVO as compared to those with CRVO, at each time point. This confirms previous findings [17, 19] and emphasizes that considerably more severe retinal damage may be caused by a more extensive impairment in retinal vein circulation.

The adverse events found in our population were consistent with the well-established safety profile of Ranibizumab and dexamethasone. The percentage of transient increase in IOP (29.1%) was similar to that reported in the previous studies [6, 17, 20, 21]. Similarly, the surgery for glaucoma has been previously described after single or multiple Ozurdex injections [21, 22]. In our affected patient, the ocular hypertension completely and stably regressed after trabeculectomy, and considering the entire FU period of 43 months, intravitreal injections still resulted in a consistent improvement in visual acuity, suggesting that the treatment was beneficial despite the adverse event.

In our population, one patient developed endophthalmitis. This is a rare complication of Ozurdex injections, whose incidence is variable in the previous literature [6, 23, 24]. Stem et al. [25] reported that the endophthalmitis rate in 3593 Ozurdex injections, over a 3-year FU period, was 0.14% of injections and 0.4% of patients (5/1051 cases). One patient in their study developed endophthalmitis twice; however, two patients continued to receive Ozurdex after the endophthalmitis with no additional adverse events. The authors concluded that endophthalmitis is an uncommon complication following Ozurdex injection that requires prompt treatment and suggested that vitrectomy with the removal of the dexamethasone implant may not be necessary in all patients. Our case confirms that there is not always a need for implant removal in order to reach a complete resolution of the infection.

The main strength of the present study is its reporting of long-term outcomes among a large patient cohort and its analysis of factors that may have an influence on visual recovery. However, a limitation of the study is its retrospective nature. In addition, the real-life clinical setting may have influenced the outcome and number of treatments as it did not allow for the strict exclusion criteria and scheduling of visits and treatments as in clinical trials. However, the aim of this study was to be representative of a typical real-world clinical experience. Although clinical trials support intravitreal therapy for RVO treatment, it is well known that visual outcomes may differ remarkably in a real-life setting. Intravitreal therapy may present a relevant burden to patients and healthcare professionals in routine clinical practice as intensive treatment and monitoring are required over long FU periods. Our findings of good long-term visual gains that are achievable in routine clinical practice among real-world individuals encourage the continuation of efforts to pursue better outcomes in the treatment of this debilitating retinal pathology.

In conclusion, the results of the present study show good long-term anatomic and functional responses to intravitreal therapy for RVO-related ME in a real-life clinical setting with a progressive reduction in the frequency of treatments. These findings support this treatment in populations with similar characteristics in similar settings.

## Figures and Tables

**Table 1:** Characteristics of the study population.

	Number eyes (%)	Age mean (SD)	Gender (%)	Phakic/pseudophakic	Ischemic (%)	Baseline BCVA logMar-mean (SD)	Baseline CMT*μ*m-mean (SD)
Whole population	223	68.2 (12.7)	116 M(52.0) 107 F (47.9)	184/39	123 (55.2%)	0.79 (0.71)	615.7 (257.5)
BRVO	124 (55.6%)	69.2 (10.6)	68 M (54.8) 56 F (45.2)	105/19	63 (50.8%)	0.60 (0.53)	536.9 (212.5)
CRVO	99 (44.4%)	66.9 (14.8)	48 M (48.5%) 51 F (51.5%)	79/20	60 (60.6%)	1.03 (0.84)	714.4 (275.4)

**Table 2:** Mean BCVA and CMT and their changes at each annual time point for the whole population and for BRVO/CRVO subgroups (results are reported until the eighth year; subsequent FU is not included because of the small sample size).

		Number of eyes	BCVA logMar-mean (SD)	BCVA improvement logMar-mean (SD)	*p* value	CMT *μ*mmean (SD)	CMT improvement *μ*m-mean (SD)	*p* value
Baseline	Whole population	223	0.79 (0.71)	—	—	615.7 (257.5)	—	—
	BRVO	124	0.60 (0.53)	—	—	536.9 (212.5)	—	—
	CRVO	99	1.03 (0.84)	—	—	714.4 (275.4)	—	—

1 year	Whole population	223	0.62 (0.68)	0.16 (0.61)	<0.0001	401.04 (183.8)	214.6 (269.6)	<0.0001
	BRVO	124	0.43 (0.51)	0.17 (0.50)	<0.0001	355.2 (135.0)	181.7 (211.5)	<0.0001
	CRVO	99	0.88 (0.78)	0.16 (0.72)	0.0169	458.5 (218.3)	255.9 (324.7)	<0.0001

2 years	Whole population	189	0.63 (0.71)	0.12 (0.68)	0.009	376.4 (171.2)	229.0 (289.1)	<0.0001
	BRVO	114	0.40 (0.47)	0.17 (0.51)	0.0002	348.7 (139.7)	183.3 (243.5)	<0.0001
	CRVO	75	0.99 (0.85)	0.03 (0.87)	0.3652	418.1 (203.9)	297.9 (336.9)	<0.0001

3 years	Whole population	147	0.65 (0.73)	0.04 (0.70)	0.2588	367.1 (171.1)	232.1 (288.6)	<0.0001
	BRVO	94	0.47 (0.55)	0.10 (0.60)	0.0522	337.9 (147.9)	193.1 (236.6)	<0.0001
	CRVO	53	0.96 (0.89)	−0.07 (0.95)	0.3089	419.9 (197.4)	302.6 (356.1)	<0.0001

4 years	Whole population	115	0.60 (0.75)	0.06 (0.74)	0.1761	379.8 (196.2)	215.9 (273.1)	<0.0001
	BRVO	73	0.39 (0.48)	0.16 (0.48)	0.0029	340.8 (158.6)	181.7 (239.2)	<0.0001
	CRVO	42	0.96 (0.97)	−0.09 (1.04)	0.2726	449.0 (236.2)	276.6 (319.1)	<0.0001

5 years	Whole population	80	0.48 (0.62)	0.14 (0.58)	0.0183	344.6 (156.4)	241.5 (251.2)	<0.0001
	BRVO	52	0.40 (0.63)	0.08 (0.51)	0.1338	330.2 (154.3)	170.8 (201.8)	<0.0001
	CRVO	28	0.61 (0.59)	0.25 (0.69)	0.0341	369.8 (159.5)	365.8 (283.1)	<0.0001

6 years	Whole population	61	0.46 (0.57)	0.19 (0.59)	0.0079	332.8 (154.0)	255.5 (255.9)	<0.0001
	BRVO	41	0.32 (0.48)	0.20 (0.48)	0.0068	313.2 (149.4)	223.6 (227.1)	<0.0001
	CRVO	20	0.76 (0.64)	0.17 (0.78)	0.1676	374.1 (159.4)	322.6 (303.6)	0.0001

7 years	Whole population	39	0.57 (0.80)	0.06 (0.78)	0.3264	306.9 (147.0)	239.2 (271)	<0.0001
	BRVO	28	0.31 (0.45)	0.21 (0.49)	0.0154	298.5 (159.1)	221.8 (241.3)	<0.0001
	CRVO	11	1.23 (1.09)	−0.34 (1.19)	0.1824	334.8 (98.9)	297 (364.4)	0.0201

8 years	Whole population	19	0.48 (0.71)	0.2 (0.52)	0.0562	270.5 (155.0)	305.1 (305.2)	0.0001
	BRVO	16	0.30 (0.51)	0.23 (0.56)	0.0616	267.2 (163.5)	257.9 (246.2)	0.0002
	CRVO	3	1.40 (0.98)	0.03 (0.06)	0.2113	300.5 (0.71)	729.5 (581.9)	0.1635

Final visit	Whole population	223	0.69 (0.80)	0.11 (0.76)	0.0175	355.6 (171.9)	260.1 (292.7)	<0.0001
	BRVO	124	0.42 (0.57)	0.19 (0.60)	0.0003	316.0 (137.1)	220.9 (246.6)	<0.0001
	CRVO	99	1.02 (0.91)	0.01 (0.91)	0.4634	405.1 (197.2)	309.3 (336.8)	<0.0001

**Table 3:** Mean visual and anatomic outcomes at each time point in BRVO versus CRVO eyes.

		BRVO	CRVO	*p* value
Baseline	Number of eyes	124	99	
	BCVA_logMar-mean-(SD)_	0.60 (0.53)	1.03 (0.84)	<0.0001
	CMT_*μ*m-mean-(SD)_	536.9 (212.5)	714.4 (275.4)	<0.0001

1 year	Number of eyes	124	99	
	BCVA_logMar-mean-(SD)_	0.43 (0.51)	0.88 (0.08)	<0.0001
	CMT_*μ*m-mean-(SD)_	355.2 (135)	458.5 (218.3)	<0.0001

2 years	Number of eyes	114	75	
	BCVA_logMar-mean-(SD)_	0.40 (0.47)	0.99 (0.85)	<0.0001
	CMT_*μ*m-mean-(SD)_	348.7 (139.7)	418.1 (203.9)	0.0057

3 years	Number of eyes	94	53	
	BCVA_logMar-mean-(SD)_	0.47 (0.55)	0.96 (0.89)	0.0003
	CMT_*μ*m-mean-(SD)_	337.9 (147.9)	419.9 (197.3)	0.0052

4 years	Number of eyes	73	42	
	BCVA_logMar-mean-(SD)_	0.39 (0.48)	0.96 (0.97)	0.0004
	CMT_*μ*m-mean-(SD)_	340.8 (158.6)	449 (236.2)	0.006

5 years	Number of eyes	52	28	
	BCVA_logMar-mean-(SD)_	0.40 (0.63)	0.61 (0.59)	0.0729
	CMT_*μ*m-mean-(SD)_	330.2 (154.3)	369.8 (159.5)	0.1426

6 years	Number of eyes	41	20	
	BCVA_logMar-mean-(SD)_	0.32 (0.48)	0.76 (0.64)	0.0051
	CMT_*μ*m-mean-(SD)_	313.2 (149.4)	374.1 (159.4)	0.0854

7 years	Number of eyes	28	11	
	BCVA_logMar-mean-(SD)_	0.31 (0.45)	1.20 (1.09)	0.0098
	CMT_*μ*m-mean-(SD)_	298.5 (159.1)	334.8 (98.9)	0.2088

8 years	Number of eyes	16	3	
	BCVA_logMar-mean-(SD)_	0.3 (0.51)	1.4 (0.98)	0.0841
	CMT_*μ*m-mean-(SD)_	267.2 (163.5)	300.5	0.1196

**Table 4:** Adverse events in the study population throughout treatment with intravitreal injections.

Adverse event	*n* (%)
Endophthalmitis	
Whole sample	1–0.4
CRVO	1–1.0
BRVO	—
Elevation in intraocular pressure^*∗*^	
Whole sample	65–29.1
CRVO	33–33.3-
BRVO	32–25.8
Surgery for refractory ocular hypertension^*∗∗*^	
Whole sample	1–0.4
CRVO	—
BRVO	1–0.8
Vascular events^*∗∗∗*^	
Whole sample	2–0.9
CRVO	2–2.0
BRVO	—
Request for emergency room service ^*∗∗∗∗*^	
Whole sample	40–17.9
CRVO	14–14.1
BRVO	26–21.0

^*∗*^Transient increase in IOP, requiring topical IOP-lowering medications; no additional procedures were required to reduce IOP. ^*∗∗*^Trabeculectomy for ocular hypertension refractory to topical medications. ^*∗∗∗*^Nonfatal myocardial infarction or nonfatal stroke. ^*∗∗∗∗*^Reasons for emergency room request: conjunctival hyperemia (*n*.5), floaters (*n*.2), feeling of a foreign body (*n*.2), and blurred vision (*n*.31).

## Data Availability

The data used to support the findings of this study include patients' age, gender, and other demographic characteristics, visual acuity measurements, central macular thickness measurements, treatments administered, clinical details on diagnosis, and safety data. They are not included in the text in order to protect patients' privacy. The anonymized data are available from the corresponding author (emi_maggio@yahoo.it) upon request or, alternatively, from the Sacro Cuore Hospital Instritutional Review Board (elvia.malo@sacrocuore.it), for researchers who meet the criteria for access to confidential data.

## Supplementary Materials

Supplementary Materials The supplementary material section includes three tables. They report, respectively, the number of eyes gaining/losing ≥1 line at each annual time point, the comparison between ischemic and nonischemic RVO outcomes, and the number of injections until the 6th year of treatment.
