# Supplementary material for: Intravitreal Injections for Macular Edema Secondary to Retinal Vein Occlusion: Long-Term Functional and Anatomic Outcomes
Source: J Ophthalmol. 2020 Feb 13;2020:7817542. doi: 10.1155/2020/7817542 (PMC7040414; doi:10.1155/2020/7817542)
Supplement: Supplementary Materials — The supplementary material section includes three tables. They report, respectively, the number of eyes gaining/losing ≥1 line at each annual time point, the comparison between ischemic and nonischemic RVO outcomes, and the number of injections until the 6th year of treatment. [file 7817542.f1.docx]

**Supplemental material**

**Table S1.** Number of eyes gaining/losing ≥ 1 line at each annual time point

| 1 year | Stable | 170 (76.2%) |
| --- | --- | --- |
|  | Gaining 1 line | 29 (13.0%) |
|  | Gaining ≥ 2 line | 6 (2.7%) |
|  | Losing 1 line | 13 (5.8%) |
|  | Losing ≥ 2 line | 5 (2.2%) |
| 2 years | Stable | 134 (70.9%) |
|  | Gaining 1 line | 26 (13.8%) |
|  | Gaining ≥ 2 line | 5 (2.6%) |
|  | Losing 1 line | 18 (9.5%) |
|  | Losing ≥ 2 line | 6 (3.2%) |
| 3 years | Stable | 105 (71.4%) |
|  | Gaining 1 line | 18 (12.2%) |
|  | Gaining ≥ 2 line | 3 (2.0%) |
|  | Losing 1 line | 13 (8.8%) |
|  | Losing ≥ 2 line | 8 (5.4%) |
| 4 years | Stable | 83 (72.2%) |
|  | Gaining 1 line | 14 (12.2%) |
|  | Gaining ≥ 2 line | 3 (2.6%) |
|  | Losing 1 line | 8 (7.0%) |
|  | Losing ≥ 2 line | 7 (6.1%) |
| 5 years | Stable | 62 (77.5%) |
|  | Gaining 1 line | 11 (13.8%) |
|  | Gaining ≥ 2 line | -- |
|  | Losing 1 line | 5 (6.3%) |
|  | Losing ≥ 2 line | 2 (2.5%) |
| 6 years | Stable | 45 (73.8%) |
|  | Gaining 1 line | 11 (18.0%) |
|  | Gaining ≥ 2 line | -- |
|  | Losing 1 line | 3 (4.9%) |
|  | Losing ≥ 2 line | 2 (3.3%) |
| 7 years | Stable | 31 (79.5%) |
|  | Gaining 1 line | 5 (12.8%) |
|  | Gaining ≥ 2 line | -- |
|  | Losing 1 line | 2 (5.1%) |
|  | Losing ≥ 2 line | 1 (2.6%) |
| 8 years | Stable | 15 (78.9%) |
|  | Gaining 1 line | 3 (15.8%) |
|  | Gaining ≥ 2 line | -- |
|  | Losing 1 line | -- |
|  | Losing ≥ 2 line | 1 (5.3%) |

**Table S2.** Comparison between ischemic and non-ischemic RVO outcomes

*Improvement in BCVA

**Improvement in CMT

|  | | BRVO | | p-value | CRVO | | p-value |
| --- | --- | --- | --- | --- | --- | --- | --- |
|  |  | Ischemic | Non-ischemic |  | Ischemic | Non-ischemic |  |
| 1 year | n. of eyes | 63 (50.8%) | 61 (49.2%) |  | 60 (60.6%) | 39 (39.4%) |  |
|  | Delta BCVA* | 0.07 (0.55) | 0.28 (0.43) | 0.0117 | 0.17 (0.79) | 0.12 (0.59) | 0.3582 |
|  | Delta CMT** | 203.5 (234.1) | 159.2 (184.6) | 0.1218 | 314.2 (339.3) | 166.2 (281.9) | 0.0103 |
| 2 year | n. of eyes | 59 | 55 |  | 48 | 27 |  |
|  | Delta BCVA | 0.10 (0.48) | 0.25 (0.53) | 0.0614 | 0.06 (0.96) | 0.08 (0.71) | 0.3528 |
|  | Delta CMT | 194.3 (280.8) | 171.8 (198.7) | 0.3113 | 330.5 (383.9) | 239.9 (226.2) | 0.1014 |
| 3 year | n. of eyes | 50 | 44 |  | 36 | 17 |  |
|  | Delta BCVA | 0.03 (0.61) | 0.17 (0.08) | 0.1163 | 0.04 (0.98) | 0.12 (0.89) | 0.3813 |
|  | Delta CMT | 200.2 (278.2) | 185.0 (181.2) | 0.3759 | 352.1 (375.72) | 191.3 (287.6) | 0.0499 |
| 4 year | n. of eyes | 38 | 35 |  | 28 | 14 |  |
|  | Delta BCVA | 0.07 (0.51) | 0.25 (0.43) | 0.0575 | 0.10 (1.15) | 0.09 (0.82) | 0.4750 |
|  | Delta CMT | 176.2 (277.3) | 187.6 (193.3) | 0.4206 | 320.4 (329.1) | 195.3 (293.6) | 0.1136 |
| 5 year | n. of eyes | 28 | 24 |  | 18 | 10 |  |
|  | Delta BCVA | 0.04 (0.55) | 0.23 (0.44) | 0.0247 | -0.36 (0.61) | -0.05 (0.81) | 0.1491 |
|  | Delta CMT | 137 (224) | 208.8 (170.1) | 0.1003 | 396.6 (310.39) | 307.2 (225.6) | 0.1915 |
| 6 year | n. of eyes | 20 | 21 |  | 11 | 9 |  |
|  | Delta BCVA | 0.13 (0.52) | 0.25 (0.45) | 0.2154 | -0.37 (0.67) | 0.06 (0.87) | 0.1222 |
|  | Delta CMT | 218.9 (272.5) | 228.7 (170.9) | 0.4455 | 321.5 (340.1) | 324.4 (253.8) | 0.4916 |
| 7 year | n. of eyes | 16 | 12 |  | 7 | 4 |  |
|  | Delta BCVA | 0.11 (0.57) | 0.35 (0.34) | 0.0953 | -0.04 (0.23) | 1.0 (1.9) | 0.1800 |
|  | Delta CMT | 242.7 (273.9) | 200.9 (211.3) | 0.3220 | 415.8 (383.0) | 59.3 (193.4) | 0.0483 |
| 8 year | n. of eyes | 9 | 7 |  | 2 | 1 |  |
|  | Delta BCVA | 0.17 (0.72) | 0.29 (0.27) | 0.3343 | -- | -- | -- |
|  | Delta CMT | 260.7 (259.7) | 254.5 (246.1) | 0.4796 | -- | -- | -- |

**Table S3**

Number of injections until the 6^th^ year of treatment

|  | Ranibizumab n. - (SD) | Ozurdex n. - (SD) |
| --- | --- | --- |
| 1 year | 4.1 (2.1) | 1.5 (0.6) |
| 2 years | 3.0 (1.1) | 0.62 (0.9) |
| 3 years | 1.2 (1.8) | 0.5 (0.9) |
| 4 years | 0.8 (1.5) | 0.5 (0.8) |
| 5 years | 1.0 (1.8) | 0.5 (0.7) |
| 6 years | 0.9 (1.8) | 0.2 (0.6) |
